# Outcomes and quality of life after major bile duct injury in long-term follow-up

**DOI:** 10.1007/s00464-020-07726-x

**Published:** 2020-06-22

**Authors:** Hanna Koppatz, Ville Sallinen, Heikki Mäkisalo, Arno Nordin

**Affiliations:** 1 Department of Abdominal Surgery, Helsinki University Hospital and University of Helsinki, Haartmaninkatu 4, 00029 Helsinki, Finland; 2 Department of Transplantation and Liver Surgery, Helsinki University Hospital and University of Helsinki, Helsinki, Finland

**Keywords:** Iatrogenic bile duct injury, Hepaticojejunostomy, Quality of life, Strasberg classification

## Abstract

**Introduction:**

Recently new standards for reporting outcomes of bile duct injury (BDI) have been proposed. It is unclear how these treatment outcomes are reflected in quality of life (QOL). The aim of this study was to report outcomes and QOL after repair of major BDI and compare repairs by hepatobiliary surgeon to repairs by non-hepatobiliary surgeons.

**Methods:**

This was a retrospective study of patients treated for major (Strasberg E-type) BDI after cholecystectomy at a tertiary hepatobiliary center. Outcomes were assessed using Cho-Strasberg proposed standards. QOL was assessed using Short Form Health Survey (SF-36) and the gastrointestinal QOL-index (GIQLI). Patients undergoing uneventful cholecystectomy matched by age, urgency, and duration of follow-up were used as controls.

**Results:**

Fifty-two patients with major BDI treated between 2000 and 2016 were included (42% male, median age 53 years). Thirty-seven (71%) patients attained primary patency (29 (83%) if primarily operated by a hepatobiliary surgeon). Actuarial primary patency rate (grade A result) at 1, 3, and 5 years was 58%, 56%, and 53% in the whole cohort, and 83%, 80%, and 80% in patients primary treated by a hepatobiliary surgeon, respectively. At 3-year follow-up 6 (11.5%) patients obtained grade B, 10 (19.2%) grade C, and 7 (13.5%) grade D result. QOL was similar in patients with BDI and controls (median SF-36 physical component 51.7 and 53.6, *p* = 1.0, mental component 53.3 and 53.4, *p* = 1.0, GIQLI 109.0 and 123.0, *p* = 0.174, respectively) at median 90 (IQR 70–116) months from cholecystectomy. QOL was similar regardless of outcome grade.

**Conclusion:**

First attempt to repair a severe BDI should be undertaken by a hepatobiliary surgeon. However, long-term QOL is not affected even by severe BDI, and QOL is not associated with the grade of the outcome.

Laparoscopic cholecystectomy (LCC) is the gold standard to manage symptomatic and complicated gallbladder stone disease, but carries a small, but significant risk of bile duct injury (BDI) [1–4]. Historically, open cholecystectomy carried a lower risk of BDI [5], but nowadays open cholecystectomy is only reserved for the most complicated cases (or after conversion from LCC) and risk of BDI after laparoscopic cholecystectomy has decreased since the introduction of LCC in the middle 80s from 0.69% to current 0.22% [6, 7].

BDI can be managed with surgical, endoscopic, or radiologic techniques depending on severity of the injury. There are several grading systems for the severity of BDI, Strasberg classification being the most commonly used [8, 9]. In Strasberg classification, E-type BDI refers to either transection or stricture of the hepatic duct(s) and are considered as major BDI [10]. Minor BDIs are usually successfully treated endoscopically, but major BDI usually requires surgery. The surgical approach can range from suturing the injury to liver transplantation but most common operation is hepaticojejunostomy (HJ) [3, 11]. Surgical BDI reconstructions are recommended to be performed in specialized centers to increase the patency of biliary tree [12, 13].

Although BDIs have been consistently classified using Strasberg classification for nearly two decades, the outcome reporting has not been standardized much due to the lack of uniform agreement on the grading of various outcomes and this has led to difficulties in comparing different series to one another [10, 14]. Moreover, the effect of BDI on quality of life (QOL) has been controversial, which might stem from lack of standardized outcome reporting [15–21].

Recently, a standardized way of reporting outcomes is proposed [10]. The aim of this study was to report both short- and long-term outcomes as well as QOL after major (Strasberg E-type) BDI in a major tertiary center using the proposed reporting standard.

## Methods

This was a retrospective review of patients surgically treated for major BDI after cholecystectomy in an academic teaching hospital (Helsinki University Hospital, HUH) that functions both as a secondary referral center for 1.2 million inhabitants and tertiary referral center for 1.9 million inhabitants. In addition, HUH being the largest hepatobiliary surgery center in Finland, and the liver transplantation unit of the whole country, patients with the most severe BDI are referred also from all other university hospitals’ areas. Patients were identified from a prospectively maintained database consisting on patients treated for BDI after cholecystectomy. Patients who had undergone a surgical operation due to BDI after cholecystectomy during 2000–2016 were included. Patients whose BDI was treated solely by endoscopic or radiologic means were excluded. In order to focus on major BDI, patients that did not have Strastberg type E BDI were excluded. Data regarding patient demographics, characteristics of the cholecystectomy and repair of BDI, as well as long-term patency of the biliary tree and late complications were manually extracted from the patient records.

In order to comprehend the QOL of patients with BDI, patients who had undergone cholecystectomy without BDI (defined as “controls”) were also recruited for QOL assessment for control purposes. Controls were searched from the HUH’s electronic operation room logs using Nordic Medico-Statistical Committee (NOMESCO) Classification of Surgical Procedures-codes JKA20 (open cholecystectomy) and JKA21 (laparoscopic cholecystectomy) limited to years 2000–2016. Each living patient with BDI was matched with three controls with closest age (up to ten-year difference), sex, duration of follow-up (up to three-month difference), and urgency of operation (emergency or elective).

QOL was assessed using Short Form Health Survey (SF-36) and the gastrointestinal QOL-index (GIQLI) questionnaires. SF-36 is widely used, tested and standardized to the Finnish population as a health status questionnaire that consists of eight different domains to evaluate mental and physical QOL [22]. SF-36 scores are standardized to range 0 to 100, where higher scores represent better health. All subscale scores were thereafter standardized using linear *z*-score transformation to perform a count to use to constitute mental component summarys (MCS) and physical component summarys (PCS) [23]. GIQLI is a disease-specific questionnaire containing measures of overall QOL, specifically emphasizing gastrointestinal symptoms. The validity and reliability of GIQLI in patients that have undergone cholecystectomy have been evaluated as high [24]. Both surveys are translated into Finnish and Swedish, which are the official languages of Finland. Along with these QOL questionnaires, patients were asked to fill out an additional questionnaire, which inquired patients regarding their education, working status, opinion of the given information before the LCC and possible discomfort and medications after the primary operation. In addition, patients with BDI were asked regarding the injury: the decision of Finnish Patient Insurance Centre, if available and the incidence of cholangitis.

The questionnaires were send up to three times to both patients with BDI and controls in cases where the first or second contact did not result in an answer. The questionnaires were posted during April 2017–January 2018. As the patients had been treated within a time-frame of 17 years, the length of follow-up varied.

The last follow-up date was defined as the last contact registered in the patient records, death, or questionnaire response date. Survival status and possible date of death were obtained from electronic patient record system, which automatically updates it from Population Register Centre, which is an extremely reliable source of survival status.

The types of BDI are reported using Strasberg classification [8] as well as newer Cho-Strasberg classification [10], which is based on the Strasberg classification. In the Cho-Strasberg classification, Grade 1 refers to Strasberg type A BDI, Grade 2 refers to Strasberg type B, C and E1-3 BDI and Grade 3 refers to Strasberg type E4 and E5 BDI [10].

The outcomes of BDI repairs are reported by the standards recently proposed by Cho et al. [10]. Shortly, patency is defined as short- and long-term continuity of the biliary tree without stents, biliary fistula, or episodes of cholangitis or jaundice after surgical reconstruction. The operation which primarily aimed to return patency was defined as index treatment. If a patient achieved the patency during 90 days after (surgical) index treatment (termed as achieving primary patency) and maintained it thereafter, the result was graded as Grade A result. If primary patency was not achieved or it was lost, the results were classified as Grade B, C, or D result depending whether they attained secondary patency. Briefly, Grade B result means that secondary patency was achieved and maintained, no stent(s) were longer than 18 months, endoscopic or radiological intervention for biliary tree was required within one year, there were up to two successfully treated cholangitis, up to one successfully treated liver abscess, and/or biliary fistula had occurred and healed within one year. Grade C result means that secondary patency was achieved and maintained, stent(s) were longer than 18 months but less than 24 months, endoscopic or radiological intervention for biliary tree was required more than once or over 1 year after the index treatment, there were three or more cholangitis, more than one liver abscess, biliary fistula had occurred and healed within two years, and/or second surgical bile duct reconstruction was performed. Grade D result means that stent(s) were present over 24 months, third surgical repair was performed, liver resection or transplantation was performed for unreconstructable biliary tree, biliary fistula was present more than 2 years, and/or liver cirrhosis developed. If the patient die2 during the index treatment period, the patency cannot be achieved. Complications 30 days after the primary repair of biliary tree continuity (defined as index treatment) are reported.

This study was approved by the institutional review board and the ethical board of HUH. Patients in QOL analyses provided written informed consent, but for other patients consent was not needed for retrospective analyses.

### Statistical analysis

The Mann–Whitney *U*-test was used to compare continuous non-normally distributed variables between groups. *p*-values < 0.05 were considered significant. In SF-36 questionnaire, up to two missing answers were imputed with the item median, but in case of more missing answers, the subject was excluded from the study. The subject was also excluded if more than three answers were missing in GIQLI questionnaire, otherwise filled with item median. Actuarial primary patency rate (i.e., Grade A result) was calculated as a Kaplan–Meier survival curve, estimates are given at 1, 3, and 5 years follow-up and the log-rank test was used for comparison of groups. Grade B, C, and D results were measured as actual rather than actuarial and reported at 3 years from completion of treatment. The statistical analyses were performed with SPSS ® version 25 (IBM, Amonk, NY).

## Results

### Patients

**Fig. 1:**
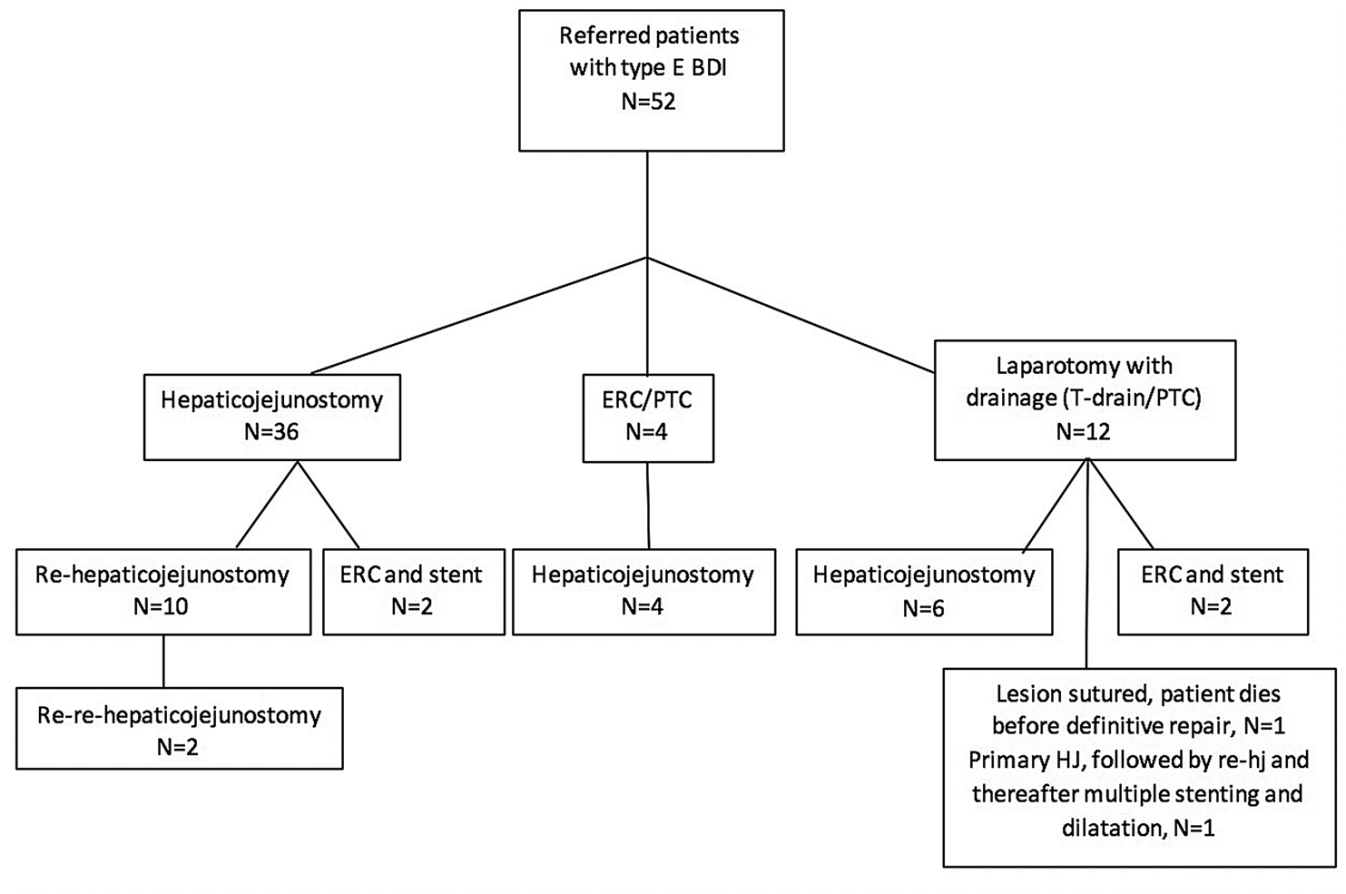
Flowchart of primary and secondary operations after bile duct injury. *BDI* bile duct injury, *ERC* endoscopic retrograde cholangiography, *HJ* hepaticojejunostomy, *PTC* percutaneous transhepatic catheter

A total of 52 patients were referred for Strasberg type E BDI during 2000–2016 (Fig. 1). Basic characteristics of the patients are shown in Table 1. Briefly, median age was 53 years, majority of cholecystectomies were converted to open surgery and performed electively and most common indication was symptomatic gallbladder stone disease. Conversion was due to noted BDI in 19 patients, bleeding in 4 patients, unclear anatomy in 6 patients, and difficult adhesions in 1 patient.

**Table 1:** Basic patient, sentinel cholecystectomy, educational and occupational characteristics

	Patients with BDI	Patients with BDI, who responded to QOL-questionnaire	Controls
	*N* = 52	*N* = 35	*N* = 53
Age^a^, years, median (IQR)	53.0 (44.3–66.4)	51.7 (45–65.2)	55.1 (44.8–67.6)
Sex, *n* (%)			
Male	22 (42.3%)	10 (28.6%)	15 (28.3%)
Female	30 (57.7%)	25 (71.4%)	38 (71.7%)
Cholecystectomy indication, *n* (%)			
Symptomatic cholecystolithiasis	26 (50%)	20 (57.1%)	30 (56.6%)
Acute cholecystitis	7 (13.5%)	4 (11.4%)	11 (20.8%)
Prior cholecystitis	13 (25.0%)	10 (28.6%)	4 (7.5%)
Choledocholithiasis	3 (5.8%)	1 (2.9%)	3 (5.7%)
Pancreatitis	1 (1.9%)	0	5 (9.4%)
Other	2 (3.8%)	0	0
Operative approach, *n* (%)			
Laparoscopic	21 (37.7%)	16 (42.9%)	50 (94.3%)
Converted	30 (57.4%)	20 (57.1%)	1 (3.8%)
Open	1 (4.9%)	0	2 (3%)
Level of urgency, *n* (%)			
Elective	42 (80.2%)	29 (82.9%)	42 (79.2%)
Emergent	10 (19.2%)	6 (17.1%)	11 (20.8%)
Primary surgeon, *n* (%)			
Resident	5 (13.1%)	5 (14.3%)	24 (45.3%)
Attending	47 (86.9%)	30 (85.7%)	29 (54.7%)
Follow up, months, median (IQR)	87.3 (64.6–121.1)	90.0 (69.4–101.2)	90.8 (68.4–120.2)
Education, *n* (%)	N/A		
No education^b^		12 (34.3%)	11 (20.8%)
Second grade education^c^		13 (37.1%)	29 (54.7%)
Academic education		10 (28.6%)	13 (24.5%)
Working status at the time of QOL questionnaires, *n* (%)	N/A		
Employed		18 (51.4%)	17 (32.1%)
Unemployed		1 (2.9%)	4 (7.5%)
Student		0	1 (1.9%)
Pensioned		16 (45.7%)	31 (58.5%)
Working ability after cholecystectomy, *n* (%)	N/A		
Working normally		24 (68.6%)	41 (77.4%)
Not capable of working		2 (5.7%)	2 (3.8%)
Was retired or unemployed		9 (25.7%)	10 (18.9%)
Need for medications after cholecystectomy (occasional or continuous), *n* (%)	N/A		
Pain killers		3 (8.6%)	3 (5.7%)
Antibiotics (occasional need)		1 (2.9%)	0
Other		5 (14.3%)	2 (3.8%)

*BDI* bile duct injury, *IQR* interquartile range

^a^At the day of the cholecystectomy

^b^Elementary school

^c^Including college and vocational education

The operative approach for the sentinel cholecystectomy in patients with BDI was laparoscopic, except for one patient, who was operated directly open because of simultaneous colon resection for colon cancer. Of the 52 patients with BDI, 46 were alive at the time when questionnaires were sent. Of these 46 patients, 35 (76%) returned adequately filled questionnaire. The questionnaires were send for 103 matched controls, of whom 53 (51%) returned adequately filled questionnaire. For SF-36, one missing answer was imputed in 5 patients and two missing answers in 1 patient. For GIQLI, one missing answer was imputed in 4 patients, two missing answers in 3 patients, and three missing answers in 1 patient. Values were missing equally in both groups. The basic characteristics and follow-up time were highly similar between all patients with BDI compared to either patients with BDI who responded to QOL questionnaires or controls (Table 1).

### Type of BDI

The characteristics of BDI are described in Table 2. The injury was identified intraoperatively in 29 (56%) patients, and most of the patients (83%) had grade 2 (Type E1-E3) injury (Table 2).

**Table 2:** Characteristics of the bile duct injury and graded outcome

	Patients with BDI	Patients with BDI, who responded to QOL-questionnaire
	(*N* = 52)	(*N* = 35)
Time of injury diagnosis, *n* (%)		
Intraoperative	29 (55.8%)	19 (54.3%)
Postoperative		
1st–2nd POD	6 (11.5%)	3 (8.6%)
3rd POD—1 week	12 (23.1%)	9 (25.7%)
More than 1 week	5 (9.6%)	4 (11.4%)

	*N* = 52	*N* = 35
BDI subclassification, *n* (%)		
E1	19 (36.5%)	13 (37.1%)
E2	18 (34.6%)	11 (31.4%)
E3	6 (11.5%)	4 (11.4%)
E4	8 (15.4%)	6 (17.1%)
E5	1 (1.9%)	1 (2.9%)
BDI grade^a^, *n* (%)		
1	0	0
2	43 (82.7%)	28 (80%)
3	9 (17.3%)	7 (20%)
Patency grading at 3-year follow-up, *n* (%)		
A	28 (53.8%)	17 (48.6%)
B	6 (11.5%)	5 (14.3%)
C	11 (21.2%)	9 (25.7%)
D	7 (13.5%)	4 (11.4%)
Patency grading at 3-year follow-up after primary HB-surgeon repair, *n* (%)		
A	28 (80.0%)	17 (81.0%)
B	2 (5.7%)	2 (9.5%)
C	2 (5.7%)	2 (9.5%)
D	3 (8.6%)	0

*BDI* bile duct injury, *HB* hepatobiliary, *QOL* quality of life, *POD* post operative day

^a^According to Cho et al. [10]

### Treatment

Primary treatment was hepaticojejunostomy for 36 (69%) patients, endoscopic retrograde cholangiography (ERC) or percutaneous transhepatic catheter (PTC) for 4 (8%) patients, and operative drainage with T-drain or operatively inserted PTC for 12 (23%) patients (Fig. 1). Eventually, 47 (90%) patients were treated with hepaticojejunostomy (Fig. 1). Twelve patients required a second hepaticojejunostomy and two patients required a third hepaticojejunostomy. The remaining five patients (10%) not treated with hepaticojejunostomy were all surgically treated in addition to endoscopic or radiological therapy (Fig. 1). None of the patients needed liver transplantation.

### Outcomes

**Fig. 2:**
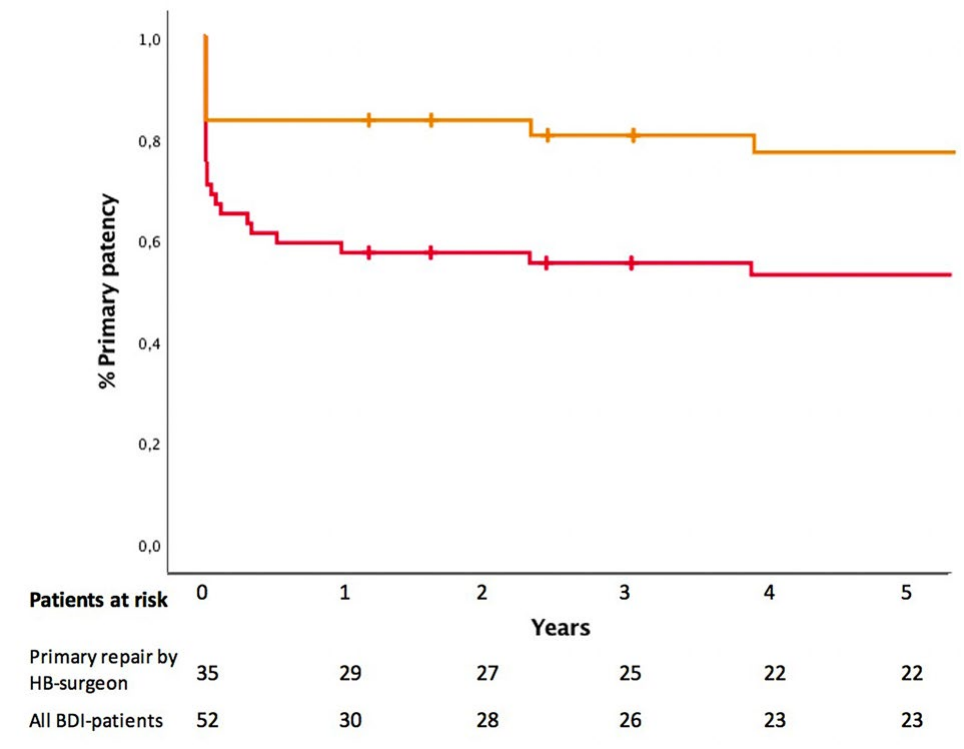
Primary patency for patients treated primarily by hepatobiliary surgeon (orange) and all patients with Strasberg type E bile duct injury (red). The actuarial long-term primary patency after 1, 3 and 5 years were 83%, 80%, and 76% for repairs by hepatobiliary surgeon and 58%, 57% and 53% for all repairs, respectively. *HB* hepatobiliary

Thirty-seven (71%) patients achieved primary patency. Primary patency was achieved in 83% (29 out of 35 patients) if primarily repaired by a hepatobiliary surgeon. In 17 patients, primary repair (15 HJ and 2 end-to-end suture) was attempted by a non-hepatobiliary surgeon, and eight of these obtained primary patency. Kaplan–Meier based actuarial primary patency rate (i.e., Grade A result) was 58%, 56%, and 53% at 1, 3, and 5 years for all patients (Fig. 2). Actuarial primary patency rates were 83%, 80%, and 80% at 1, 3, and 5 years if the first repair was performed by a hepatobiliary surgeon (Fig. 2). Fifteen patients did not achieve primary patency for the following reasons: a drain or stent was held over 90 days after index treatment in seven patients (stent or PTC four, intra-abdominal drain in two and T-drain in one patient), re-repair was performed during the index treatment period in four patients, liver resection was performed within the index treatment period in two patients, and two patients died during the index treatment period. Nine patients lost the achieved primary patency for developing cholangitis (*N* = 7) or jaundice (*N* = 2) caused by anastomosis stricture. One of these patients lost the primary patency after four years. Seven out of these nine patients, who had lost primary patency, achieved secondary patency.

**Fig. 3:**
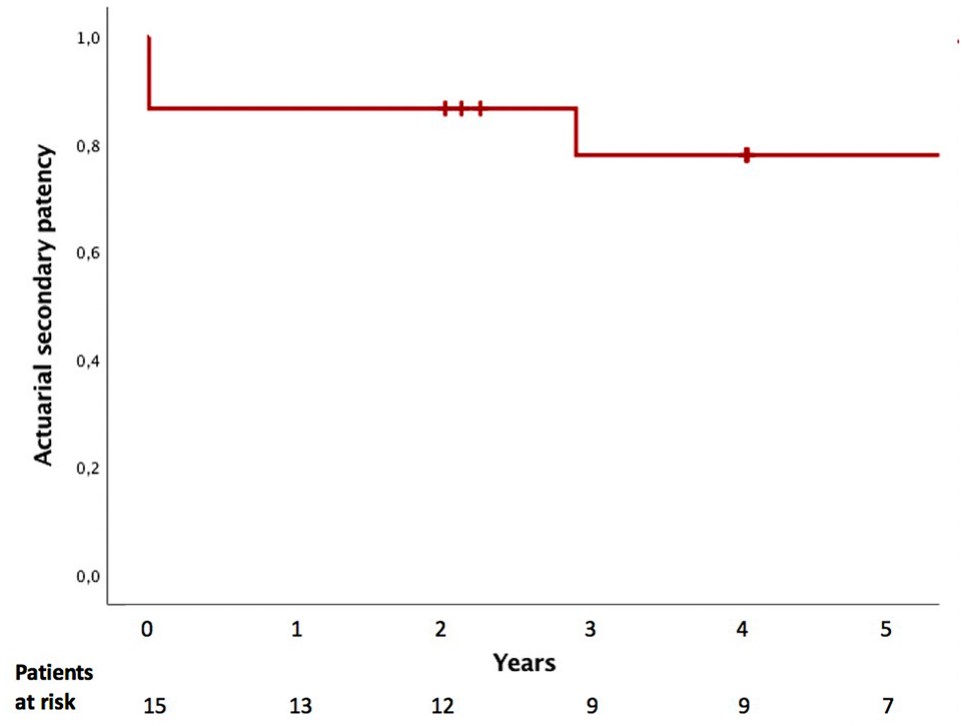
Patency after secondary re-repair with hepaticojejunostomy. The actuarial secondary patency rate after 1, 3, and 5 years were 87%, 78%, and 78%

Ten out of fifteen patients, who did not achieve primary patency, achieved secondary patency. Thus, in total, seventeen patients achieved secondary patency. Out of these seventeen patients, six patients had Grade B result, ten had grade C result, and one had grade A result after at 3-year follow-up (the patency was lost after 3 years). Additionally, seven patients had Grade D result for the following reasons: two patients underwent liver resection, stent was in place for more than two years in one patient, two patients underwent a third surgical repair, and two patients died during the index treatment period. For the fifteen patients undergoing re-repair by hepaticojejunostomy, actuarial secondary patency rate at 1 year was 87% (13 patients) and 73% at the end of follow-up (median 94 months) (Fig. 3). Graded outcomes were better if the primary repair was performed by a hepatobiliary surgeon (Table 2).

Details of postoperative complications are described in Table 3. After the primary attempt to attain the continuity of the biliary tree, 22 out of 51 (43.1%) patients did not get any complications during the 30-day follow-up (Table 3). However, eight patients had more than one complication. The median length of hospital stay after primary cholecystectomy and the index treatment day were 11 (IQR 7–18) days and 9 days (IQR 6–17) days. During this 30-day period after cholecystectomy, one patient died (Clavien-Dindo grade 5). Alltogether, six patients died in the follow-up period. Three of them died because of BDI. First patient was a 73-year old woman who had undergone elective open right colon resection and cholecystectomy simultaneously. She had an E4-type BDI. Nine days after initial operation with drainage and internal stenting, a hepaticojejunostomy with a prophylactic percutaneous transhepatic drainage was performed. She experienced severe postoperative hemorrhage requiring right hepatic artery embolization and reoperation with packing and open abdomen. She developed multi-organ failure and died 34 days after hepaticojejunostomy operation. Second patient was a 90-year old man who underwent emergent laparoscopic cholecystectomy for perforated cholecystitis. He had a lesion in portal vein and E3-type BDI, which were repaired by suturation and drainage, and also underwent a reoperation for fascial rupture. He died eight days after the first repair due to sepsis and cardiac failure. Third patient was 51-year-old man who had E2-type BDI after elective laparoscopic cholecystectomy for symptomatic cholecystolithiasis. The BDI was reconstructed by hepaticojejunostomy at same operation immediately after recognized complication and primary patency was achieved. The primary patency was lost for anastomosis stricture and re-hepaticojejunostomy was performed to achieve secondary patency 155 days from hepaticojejunostomy. After 9 years, he developed worsening cholangitis, eventually liver abscesses, which he died of 9 years 8 months after the second hepaticojejunostomy. Other three patients died for reasons not related to BDI (kidney cancer, lung cancer, and multiple myeloma).

**Table 3:** Postoperative complications 30 days after the definite surgical repair

Clavien-Dindo grade	Patients with BDI^a^	Patients with BDI, who responded to QOL-questionnaire
	(*N* = 51)	(*N* = 35)
None	31 (60.8%)	22 (62.9%)
Grade 1	1 (2.0%)	0%
Postoperative hematoma without interventions	1 (2.0%)	0
Grade 2	8 (15.7%)	7 (20.0%)
Pulmonary embolism	3 (5.8%)	2 (5.6%)
Pneumonia	2 (3.9%)	2 (5.6%)
Bile leak, antibiotics	1 (2.0%)	1 (2.9%)
Hematoma infection	1 (2.0%)	1 (2.9%)
Sepsis	1 (2.0%)	1 (2.9%)
Grade 3a	8 (15.7%)	4 (11.4%)
Bile leak, percutaneous drainage	4 (7.9%)	1 (2.9%)
Intraluminal bleeding	1 (2.0%)	1 (2.9%)
Pleural drainage	3 (5.8%)	2 (5.6%)
Grade 3b	8 (15.7%)	6 (17.1%)
Re-laparotomy and drainage	1 (2.0%)	1 (2.9%)
Re-laparotomy and liver resection	2 (3.9%)	1 (2.9%)
Re-laparotomy and hepaticojejunostomy	5 (9.8%)	4 (11.3%)
Grade 4a	2 (3.9%)	2 (5.7%)
Acute kidney injury	1 (2.0%)	1 (2.9%)
Respirator ventilation need	1 (2.0%)	1 (2.9%)
Grade 4b	1 (2.0%)	1 (2.9%)
Acute kidney injury and respiratory distress syndrome	1 (2.0%)	1 (2.9%)
Grade 5 (death)	1 (2.0%)	N/A

One or more complication per patient

*N/A* not applicable

^a^One patient died before definite repair

### Quality of life

Twenty-five patients (62.9%) with BDI and 21 controls (34%) of controls did not think they had received enough information about complication risk (*p* = 0.016). Patients with BDI were more likely to experience disturbance in daily living caused by performed cholecystectomy (16 vs 8 patients; 46 vs 15%; *p* = 0.003). Patients with BDI described following symptoms: gastrointestinal problems (9 patients), stomach pain (7 patients), scar issues (7 patients) and overall fatique (3 patients). Two patients had an incisional hernia and two patients had recurrent episodes of cholangitis. Control patients complained about stomach pain (5 patients) and gastrointestinal problems (3 patients). In both groups 2 patients (BDI patients 5.7% and control patients 3.8%) reported not being able to work normally as before the initial cholecystectomy (Table 2).

**Fig. 4:**
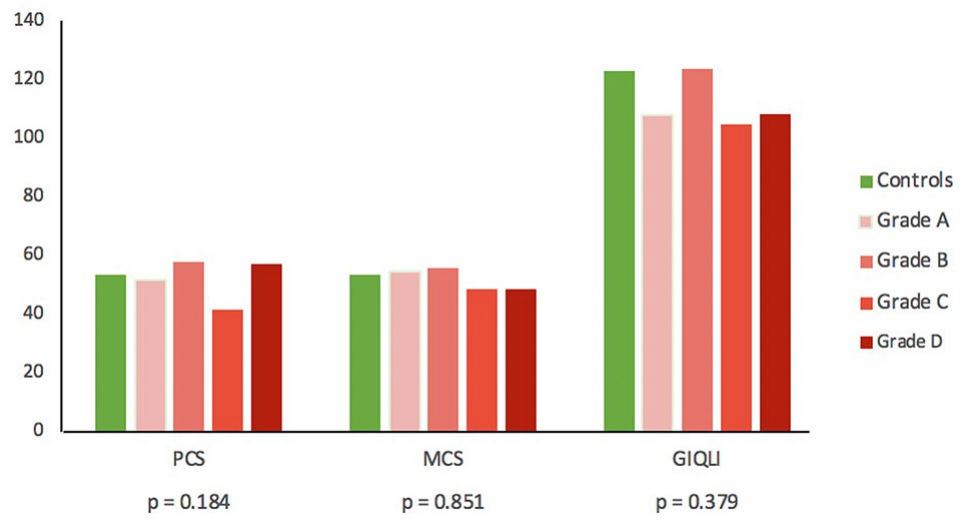
Quality of life after uncomplicated cholecystectomy (Controls) and BDI reconstruction by the BDI grading (A-D) in median 90 months (IQR 69.7–116.0) follow-up. (Median (IQR) PCS: Controls 53.4 (46.8–57.7), Grade A 51.7 (43.6–57.6), Grade B 57.8 (41–60.6), Grade C 41.4 (35.8–51.1), Grade D 57.0 (42.0–60.5). Median (IQR) MCS: Controls 53.4 (45.2–56.6), Grade A 54.4 (40.7–57.2), Grade B 55.6 (51.1–57.4), Grade C 48.6 (39.3–61.0), Grade D 48.4 (40.0–52.6). Median (IQR) GIQLI: Controls 123 (105–129.5), Grade A 108 (83.5–131.5), Grade B 124 (107–129), Grade C 105 (80.5–125.5), Grade D 108.5 (81–130)). *BDI* bile duct injury; *PCS* physical component summary, *MCS* mental component summary, *GIQLI* gastrointestinal quality-of-life index

QOL according to GIQLI and SF-36 questionnaires did not differ between patients with BDI and controls. Also, the QOL was similar between patients with different outcome grades (Fig. 4). Of 31 patients who filled a complaint to The Finnish Patient Insurance Centre, 24 (77.4%) patient’s were granted for compensation. The QoL was neither dependent on litigation decision (Negative vs favorable decision; median (IQR) PCS 41 (35.1–56.5) vs 54.6 (43.4–58.4), *p* = 0.539; MCS 54.2 (47.5–57.8) vs 53.1 (42.7–57.4), *p* = 0.909; GIQLI 100 (74–124) vs 114.5 (97–129), *p* = 0.179). Sensitivity analyses were performed by excluding the patients in whom any answers were missing in QOL questionnaires, but this did not change the result.

## Discussion

In this study, outcomes of BDI repair after cholecystectomy are reported using recently proposed new standards [10]. The repair was successful in attaining primary patency in 71%, and in 83% if done by a hepatobiliary surgeon. Grade A result (actuarial primary patency rate), i.e., obtaining patency of the biliary tree in the first operation and maintaining it thereafter, was 54% at 1-year follow-up. However, this was largely due to the fact that many patients (33%) had already undergone a repair attempt by a non-hepatobiliary surgeon before arriving to our hospital, which functions as a tertiary center for the whole country and where the most difficult BDIs are referred to. If the primary repair was performed by a hepatobiliary surgeon the rate of grade A result was 83% at 1-year follow-up. Taking this into account, it was surprising to note that in the long-term follow-up, the QOL of patients who had severe BDI after cholecystectomy is similar with patients who underwent uneventful cholecystectomy. Furthermore, QOL was affected neither by the grade of outcome nor the decision to compensate a patient claim.

The new proposed standard of outcome reporting has not yet been extensively used, and thus comparison of the outcomes between series is limited. Two reports using the proposed standards, to our knowledge, have been published in addition to the proposal manuscript [10, 25, 26]. One of these reported the outcomes from Mexico, and compared their results to outcomes in US as reported by Cho et al. [10, 26]. They reported attained primary patency rate of 83%, and 63% rate of grade A result at 10-year follow. Grade B, C, or D results were not reported, and their cohort included also patients with Strasberg type D BDI. Cuendis-Velázquez et al. published outcomes after minimally invasive BDI repair of Strasberg type E BDI in Mexico also and reported 100% attained primary patency rate and grade A result in 93% of patients undergoing laparoscopic repair at median 49-month follow-up and 100% in patients undergoing robotic repair at median 16-month follow-up. Grade B rate was 0%, grade C 8%, and grade D 0% after first year. Originally Cho et al. presented their results, where primary patency was attained in 94%, and 5- and 10-year grade A result was 92%.

QOL after repair of BDI that occurred during complicated laparoscopic cholecystectomy has been a subject of interest for decades, but the results have been contradicting. Some studies have found decrease in physical [19] and some in mental QOL [14, 18, 19], while others conclude that the QOL in the long-term follow-up is similar to patients who had undergone an uneventful cholecystectomy [17, 20]. A recent report has claimed that BDI leads to decreased working ability [27], while we found similar working ability after BDI and uneventful cholecystectomy. The major difference in our study, compared to earlier ones, was that all patients had a major, Strasberg type E, BDI. This might affect the results, and paradoxically patients who have undergone major complication, and survived it, might have higher QOL in the long run compared to patients with minor BDI.

There are limitations in this study. First, the number of patients was relatively low, and some patients were attempted a BDI repair outside the tertiary hospital by non-hepatobiliary surgeon. This was found to decrease the rates of successful repairs in our series, but it is of note that successful repairs outside our center are never referred, and thus the series includes only the failed repairs by non-hepatobiliary surgeons. Second, the series only included major BDI, defined as Strasberg type E BDI, and results must be interpreted that in mind. Third, this was a retrospective study with all inherited limitations. Fourth, QOL was assessed at various timepoints as the patients had varying follow-up at the time the questionnaires were send. This might affect the experienced QOL. However, also the control group had similarly varying follow-up period. It might be that a difference in QOL or ability to work could be found if studied in fixed timepoints closer to the event. Further, there is always a concern that the response rate of patients with BDI or uneventful cholecystectomy were not entirely random, and results might be affected by patients not responding to the query. Since the number of patients able to participate this study was low, this part of the study may not be generalizable in all respects and further research is needed.

In conclusion, BDI after cholecystectomy should be referred immediately to a tertiary hepatobiliary unit for repair in order to achieve optimal outcomes. New proposed standards for outcome reporting allow comparison between centers, but these grades of these outcomes were not reflected in QOL in long-term follow-up.
